# Embelin and levodopa combination therapy for improved Parkinson’s disease treatment

**DOI:** 10.1515/tnsci-2022-0224

**Published:** 2022-06-29

**Authors:** Vagdevi Hangarakatte Ramachandra, Senthilkumar Sivanesan, Anand Koppal, Shanmugam Anandakumar, Matthew D. Howell, Ethirajan Sukumar, Rajagopalan Vijayaraghavan

**Affiliations:** Department of Research and Development, Saveetha Institute of Medical and Technical Sciences, Chennai 602105, Tamilnadu, India; Department of Pharmacology, Subbaiah Institute of Medical Sciences and Research Centre, Shivamogga 577222, Karnataka, India; Department of Phytoinformatics, Yukai Care Solutions LLP, Chennai 600011, Tamilnadu, India; Department of Research and Development, Saveetha Institute of Medical and Technical Sciences, Chennai 602105, Tamilnadu, India; Department of Biomedical Sciences, Iowa State University, Ames, IA, 50011, United States of America

**Keywords:** gut pathology, Nurr1, oxidative stress, tyrosine hydroxylase, *in silico* docking studies

## Abstract

Parkinson’s disease (PD), a progressive neurodegenerative disorder, affects dopaminergic neurons. Oxidative stress and gut damage play critical roles in PD pathogenesis. Inhibition of oxidative stress and gut damage can prevent neuronal death and delay PD progression. The objective of this study was to evaluate the therapeutic effect of embelin or the combination with levodopa (LD) in a rotenone-induced PD mouse model. At the end of experimentation, the mice were sacrificed and the midbrain was used to evaluate various biochemical parameters, such as nitric oxide, peroxynitrite, urea, and lipid peroxidation. In the substantia nigra (midbrain), tyrosine hydroxylase (TH) expression was examined by immunohistochemistry, and Nurr1 expression was evaluated by western blotting. Gut histopathology was evaluated on tissue sections stained with hematoxylin and eosin. *In silico* molecular docking studies of embelin and α-synuclein (α-syn) fibrils were also performed. Embelin alone or in combination with LD ameliorated oxidative stress and gut damage. TH and Nurr1 protein levels were also significantly restored. Docking studies confirmed the affinity of embelin toward α-syn. Taken together, embelin could be a promising drug for the treatment of PD, especially when combined with LD.

## 1 Introduction

Parkinson’s disease (PD) is the second most common progressive, multifactorial neurodegenerative disorder after Alzheimer’s disease. It has a complex etiology and mostly affects elderly people [1]. Pathophysiologically, PD is characterized by the degeneration of midbrain dopaminergic neurons in the substantia nigra (SN) and is associated with motor dysfunctions. The neurotransmitter dopamine (DA) has a major role in movement, motivation, memory, reinforcement, motor skills, and other functions. Decreased or altered neurotransmission as a result of degeneration of dopaminergic neurons leads to cardinal features of PD, such as tremors at rest, rigidity, bradykinesia (akinesia), and postural instability [2]. PD is often associated with neuropsychiatric problems, as well as abnormalities in the olfactory, visual, somatosensory, and autonomic systems, although it is primarily a motor disorder [3]. The exact mechanism of dopaminergic neurodegeneration is not clear. Mitochondrial damage, oxidative stress, excitotoxicity, misfolding and aggregation of proteins, and impairment of protein clearance pathways may be responsible for the onset and progression of PD [4,5,6,7]. These factors are closely linked to autophagy, a highly conserved cellular catabolic process that involves elimination of cellular dysfunctional proteins and organelles. Growing evidence indicates the importance of autophagy and dysregulation in the process of neurodegeneration [8].

Dysfunctional mitochondria show a reduction in energy production and utilization. The effect of environmental toxins such as herbicides, pesticides, fungicides, and insecticides on health is a major concern [9,10]. Many studies suggest a close relationship between exposure of these toxic chemicals and the development of PD [11,12,13,14]. By products of oxidative phosphorylation, such as hydrogen peroxide and superoxide radicals, are scavenged by antioxidants in cells under normal condition. However, reactive oxygen species (ROS) levels are significantly increased in pathological conditions associated with mitochondrial dysfunction [15,16,17,18,19]. Despite brain energy dysfunction, an increase in brain urea has been unraveled in other neurodegenerative disorders, such as Alzheimer’s and Huntington diseases [20,21,22,23]. An animal model of PD also shows this increase in brain urea [24]. The transcription factor nuclear receptor related-1 (Nurr1) is critical for the homeostasis of dopaminergic neurons, because it plays a vital role in synthesis, survival, and functional integrity and also exerts protective effects against oxidative stress. Nurr1 downregulation is associated with the progressive neuropathology of dopaminergic neurons and PD pathogenesis [25,26,27]. Nurr1 plays a role in maintaining the homeostasis of nigral dopaminergic neurons as well as in situations of stress and cell damage. It is important to explore its functions in PD. Researchers have reported that the level of Nurr1 in midbrain dopaminergic neurons decreases in the elderly [28] as well as in PD [29]. One hypothesis is that hypoxia-inducible factor-1 alpha (HIF-1α) and Nurr1 are crucial for the development and survival of dopaminergic neurons, and PD pathogenesis is aggravated due to deficiencies in these proteins [30,31]. Restoring Nurr1 activity could be a useful therapeutic approach for PD [30].

The enteric nervous system (ENS), also referred to as the “second brain,” is a bidirectional integrated system that communicates extensively with the central nervous system (CNS) [32,33,34]. Recent studies on the gastrointestinal (GI) tract have emphasized the role of the ENS in the development of PD [35,36]. Researchers have reported that α-synuclein (α-syn) migrates from the GI tract to the brain [37]. Epidemiological studies have also shown a strong link between exposure to noxious environmental factors, such as heavy metals, insecticides, herbicides, and pesticides, and the evolution of neurodegenerative processes in PD by causing oxidative stress and genetic changes [38,39,40]. Generation of a massive amount of ROS as a result of oxidative stress alters proteins and lipids and disrupts homeostasis [41,42].

The current treatments available for PD are focused on symptomatic improvement. It is critical to find a potential candidate drug that targets the molecular mechanisms of PD pathogenesis to delay neurodegeneration. Natural products and their derivatives from plants are vital for novel drug development [43]. One promising plant is *Embelia ribes* Burm. f. (Family: Myrsinaceae). *E. ribes* berry extract has been extensively investigated for its various pharmacological properties, including antioxidant, anxiolytic, antidepressant, anticonvulsant, antidiabetic, and antimicrobial effects [44]. It has also tested in animal models of Alzheimer’s [45] and Huntington [46] diseases, with promising findings. The main active principal isolated from *E. ribes* berries is embelin (2,5-dihydroxy-3-undecyl-1,4-benzoquinone). This compound can cross the blood–brain barrier to promote beneficial effects on the CNS [47]. Toxicity studies of embelin in mice (50 or 100 mg kg^−1^) was found to be safer and did not change body weight [48]. Mice carrying xenograft pancreatic tumors were fed embelin (75 mg kg^−1^) for 6 weeks. It was well tolerated and showed no toxic effects [49].

The standard drug levodopa (LD) is the only approved symptomatic treatment for PD but shows several problems: “dopa-resistant” motor symptoms (postural abnormalities, freezing episodes, and speech impairment) and non-motor symptoms, such as autonomic dysfunction, mood and cognitive impairment, and/or drug-related side effects (especially psychosis, motor fluctuations, and dyskinesias) [50,51]. It would be useful to identify a treatment that could complement LD, allowing for the reduction of the LD dose while preventing the various motor and non-motor symptoms that sometimes accompany this reduction. The mechanisms concerning neurodegeneration seem to involve aggregation of the presynaptic molecule α-syn into toxic oligomers and fibrils. Hence, it would be useful to identify a treatment that could bind to α-syn, breaking up toxic aggregates or preventing aggregates from forming. This ability could ameliorate symptoms in patients with PD. Overall, the currently available PD therapy does not adequately treat the disease. The present work employed a mouse model with rotenone-induced PD symptoms to evaluate the antioxidant potential of embelin alone or in combination with LD. Furthermore, *in silico* docking studies were used to characterize how embelin binds to α-syn.

## 2 Materials and methods

### 2.1 Chemicals and reagents

Rabbit polyclonal β-actin and rabbit polyclonal Nurr1 primary antibodies were procured from Invitrogen, Thermo Fisher Scientific (Waltham, MA, USA). The main chemicals used in this study were procured from the following sources: rotenone (TCI Chemicals Pvt. Ltd., Chennai, India); the Abcam Urea Assay Kit ab83362 (Boston, MA, USA); thiobarbituric acid (TCI Chemicals Pvt. Ltd., Tamil Nadu, India); olive oil, Thuruthel Drug Lines (ERUMBA Pharmacy, Payyannur, Kerala, India); l-DOPA (product code: 77201; Sisco Research Laboratories Pvt. Ltd., Maharashtra, India); dimethyl sulfoxide (DMSO) (Pure Chemicals Co., Chennai, India); and hydroxypropyl cellulose (HiMedia, Mumbai, India). An authenticated sample of *E. ribes* (berries) was provided by Dr. V. Chelladurai, Botanist, Thirunelveli, Tamil Nadu, India. All other chemicals and reagents used were of analytical grade.

### 2.2 Preparation of rotenone

Fifty milligrams of rotenone was dissolved in 1 mL of DMSO. From this solution, 0.2 mL was mixed with 19.8 mL olive oil to prepare the stock solution.

### 2.3 Preparation of embelin

Coarsely powdered *E. ribes* berries (1.5 kg) were extracted three times with 2 L of *n*-hexane using the cold extraction method (24 h for each extraction). The solvent was decanted, pooled, and distilled in a boiling water bath. The extract was concentrated *in vacuo* and subjected to column chromatography over silica gel (100–200 mesh). Benzene was used to elute embelin from the column. This procedure yielded an orange powder that, when crystallized with diethyl ether, afforded orange plates of embelin (yield: 2 g) [52]. The structural characterization of embelin was confirmed in earlier work by Fourier transform infrared (FT-IR) spectroscopy [53].

### 2.4 Animal grouping and experimental design

Male Swiss albino mice (25–30 g) were procured from M/s Biogen Laboratory (Bengaluru, India). Animals were housed in ventilated polypropylene cages and acclimatized for 7 days to laboratory conditions before starting experiments. They were kept under ambient conditions with a natural light/dark cycle at 25 ± 2°C and 40–60% relative humidity and fed with standard pellet diet and water *ad libitum*.

The mice were randomly divided into seven groups (10 per group). The treatments were as follows: group I, vehicle control, namely olive oil (2 mL kg^−1^ orally [p.o.]); groups II–VII, rotenone (2.5 mg kg^−1^, intraperitoneally); group III, embelin (20 mg kg^−1^, p.o.); group IV, embelin (40 mg kg^−1^ p.o.); group V, embelin (20 mg kg^−1^ p.o.) and LD (7.5 mg kg^−1^ p.o.); group VI, embelin (40 mg kg^−1^ p.o.) and LD (7.5 mg kg^−1^ p.o.); and group VII, LD (7.5 mg kg^−1^ p.o.). The treatment protocol in mice as indicated above was for a period of 21 days.


**Ethical approval:** The research related to animals’ use has been complied with all the relevant national regulations and institutional policies for the care and use of animals. All animal experimental procedures were performed according to the guidelines of Committee for the Purpose of Control and Supervision of Experiments on Animals, Government of India, and were approved by Institutional Animal Ethics Committee (reference number: SU/CLAR/RD/003/2019).

### 2.5 Preparation of the PD mouse model

Previous work has shown that chronic daily intraperitoneal injection of rotenone that is prepared using a natural oil causes behavioral (locomotor) deficits and neurochemical abnormalities characteristic of PD [54,55]. Therefore, in the present work, rotenone was prepared in a mixture of olive oil and DMSO (99:1) and administered intraperitoneally to Swiss mice for 21 days.

### 2.6 Cardiac perfusion and brain homogenate preparation

At the end of the experiment, the animals were anesthetized using isoflurane and subjected to cardiac perfusion using normal saline (0.9% NaCl) to eliminate the blood. The skull of the mice was opened and the midbrain carefully isolated and washed with ice-cold phosphate-buffered saline (PBS), pH 7.4. The whole midbrain tissue isolated from each mouse was kept on ice and homogenized with 0.1 M PBS (pH 7.0) using a Potter–Elvehjem PTFE-coated pestle and glass tube (PRO Scientific Inc., Oxford, CT, USA).

### 2.7 Isolation of the postmitochondrial fraction

The midbrains were immediately immersed in 0.7 mL of ice-cold isolation buffer (IB) composed of 225 mm mannitol, 75 mm sucrose, 1 mm ethylene glycol-bis(β-aminoethyl ether)-*N,N,N′,N′*-tetraacetic acid (EGTA), 5 mm HEPES-KOH (pH 7.2), and 1 mg mL^−1^ of essential fatty acid-free bovine serum albumin (BSA) in a 5 mL plastic tube. The tissue was homogenized at 25,300*g* using a tissue homogenizer (REMI Lab Homogeniser RQ-127A/D, Maharashtra, India) and transferred into a 1.5 mL microcentrifuge tube. The brain homogenate was centrifuged at 1,100*g* for 2 min in a refrigerated centrifuge (Eppendorf, Model 5425R, Hamburg, Germany). The supernatant was placed aside and the pellet was resuspended in 0.2 mL of the buffer and centrifuged again at 1,100*g* for 2 min. The pellet was discarded and the supernatant was combined with that from the first centrifugation. Next, 0.5 mL of combined supernatants was mixed with 0.07 mL of 80 vol% Percoll solution, carefully layered on top of 0.7 mL of 10% Percoll solution, and centrifuged at 18,500*g* for 10 min. Then, 80% Percoll was diluted in buffer to prepare the 10% solution. The mitochondria-enriched fraction was collected at the bottom of the tube and resuspended in 0.7 mL of washing buffer composed of 250 mM sucrose, 5 mM HEPES-KOH (pH 7.2), 0.1 mM EGTA, and 1 mg mL^−1^ BSA. The suspension was centrifuged at 10,000*g* for 5 min. The final mitochondrial pellet was resuspended in 0.07 mL of washing buffer and stored on ice. To isolate mitochondria, the same procedure was employed as described above, except that IB was supplemented with 0.01% digitonin at the tissue homogenization step, to release mitochondria. The mitochondrial protein content was measured with a Lowry assay kit (Bio-Rad, Haryana, India) according to the manufacturer’s instructions. After removing the pellet, the postmitochondrial supernatant fraction was used to estimate the levels of nitric oxide (NO), urea, peroxynitrite, and malondialdehyde (MDA), a measure of lipid peroxidation (LPO).

### 2.8 Measurement of LPO

The MDA content was determined by the thiobarbituric acid (TBA) reaction as described previously [56]. To 0.5 mL of homogenate, 1.5 mL of 20% acetic acid, 0.2 mL of sodium dodecyl sulfate (SDS), and 1.5 mL of TBA were added. The mixture was adjusted to 4.0 mL with distilled water and then heated for 60 min at 95°C using a glass ball as a condenser. After cooling, 4.0 mL of a butanol-pyridine mixture was added and the mixture was shaken well. After centrifugation at 1,792*g* for 10 min, the organic layer was removed and its absorbance was read at 532 nm. The standards and blanks were treated in a similar manner. The MDA concentration, an indicator of LPO, is expressed as nmol mL^−1^.

### 2.9 Measurement of NO

Nitrite ( {\text{NO}}_{2}^{-}] ) and nitrate ( {\text{NO}}_{3}^{-}] ) are stable final products of NO metabolism and may be used as indirect markers for the presence of NO. The total NO concentration is commonly determined as the sum of the {\text{NO}}_{2}^{-}] and {\text{NO}}_{3}^{-}] concentrations. In the analyzed samples, {\text{NO}}_{3}^{-}] was reduced to {\text{NO}}_{2}^{-}] in the presence of cadmium and then converted to nitric acid; this process used Griess’s reagent o produce a colored product (Sigma-Aldrich, Stainheim, Germany). The {\text{NO}}_{2}^{-}] concentration was determined by spectrophotometric analysis at 540 nm [57].

### 2.10 Measurement of peroxynitrite

Peroxynitrite levels were measured according to the method described by Beckman et al. [58] Peroxynitrite-mediated nitration of phenol was measured spectrophotometrically at 412 nm. In brief, 100 µL of homogenate was placed in a glass test tube, to which 5 mM phenol in 5 M sodium phosphate buffer (pH 7.4) was added to a final volume of 2 mL. After mixing, the solution was incubated for 2 h at room temperature and then 15 µL of 0.1 M sodium hydroxide was added and the absorbance was read at 412 nm. The method is based on the oxidation of *o*-phenylenediamine, a colorless substance, by peroxynitrite to yield a colored product. The absorbance increase of this chemical reaction is linearly related to the concentration of peroxynitrite in the range of 4.4 × 10^−7^ to 8.0 × 10^−6^ mol L^−1^, with a detection limit of 1.7 × 10^−7^ mol L^−1^ (3*σ*).

### 2.11 Measurement of urea

Elevated brain urea levels in neurodegenerative Huntington’s disease patients were reported as a major metabolic defect [59]; hence, brain urea levels were measured. In a 96-well plate, a 50 μL reaction mix containing urea assay buffer (42 μL), oxiRed probe (2 μL), enzyme mix (2 μL), developer (2 μL), and converter enzyme (2 μL) was added to each well containing 50 µL of urea standards, test samples, or blank control. The reaction mixture was mixed well and incubated for 60 min at 37°C (protected from light). The absorbance at 570 nm was measured using spectrophotometer (Elico, B-200, Hyderabad, India).

### 2.12 Western blot analysis

The SN tissue homogenate was prepared in lysis buffer containing 1% protease inhibitor (HiMedia Laboratories) for 1 h at 4°C. Samples were then centrifuged at 10,000*g* for 15 min at 4°C. Supernatants were collected and the protein concentration was determined using a Bradford protein assay kit. Equal amounts of protein (40 μg) were separated on 10% acrylamide gels using SDS–polyacrylamide gel electrophoresis and electrotransferred onto a polyvinylidene difluoride membrane. Membranes were incubated with 5% nonfat milk for 1 h at room temperature (to block nonspecific protein binding) prior to incubation overnight at 4°C in a solution of rabbit polyclonal anti-β-actin (1:1,000) and rabbit polyclonal anti-Nurr1 (1:1,000). The next day, membranes were washed three times with Tris-buffered saline with 0.5% Tween 20 (TBST), incubated with goat anti-rabbit IgG conjugated to horseradish peroxidase (1:5,000) for 1 h at room temperature, and then washed three times with TBST. The target proteins were visualized using ECL reagent and exposed to a piece of X-ray film. The densities of specific protein bands were determined using Adobe Photoshop CS6 software (version 12.0). The Nurr1 (66 kDa) protein level was normalized to the β-actin (42 kDa) protein level (loading control).

### 2.13 Gut histopathology and brain immunohistochemical staining

Gut tissue was fixed in 10% neutral buffered formalin, dehydrated, embedded in paraffin, and then sectioned at a thickness of 5 μm and stained with hematoxylin and eosin (H&E) for histopathological investigation. For histomorphometry analysis, the gut tissue samples were well-oriented with longitudinally cut crypts to precisely assess alterations in the overall tissue architecture. Regarding gut tissue samples, at least 4–5 consecutive villi well distended from the base to tip were rated. The number of goblet cells per villus was counted in 10 well-oriented and adjacent crypt villi on each section stained with H&E. All measurements were made at 100× magnification. On each slide, one section that represented the best view of the villus and crypts was selected for analysis. The slides were examined under an Olympus light microscope and photomicrographs were taken with a Sony camera.

For brain tissue, mice in each group were anesthetized with isoflurane and then transcardially perfused with 0.9% normal saline to clear the blood from the brain. The brain from each mouse was immersed in 10% neutral buffered formalin (the fixative solution) for 4 h. The tissues were cryoprotected in 30% sucrose, embedded in tissue-freezing medium with liquid nitrogen, and sectioned at 3–5 µm using a cryostat. Sections were stored under anti-freeze buffer. Parallel free-floating sections were subjected to endogenous peroxidase quenching with 1% hydrogen peroxide in PBS, followed by treatment with blocking buffer (5% normal chicken serum and 0.3% Triton X-100 in PBS overnight at 4°C) to block nonspecific protein binding. Sections were incubated overnight at 4°C with mouse monoclonal anti-tyrosine hydroxylase (TH; diluted 1:3,000; Thermo Fisher Scientific, Invitrogen). After washing with PBS, tissues were incubated for 2 h at room temperature with affinity-purified rabbit anti-mouse IgG antibody (diluted 1:50 dilution; Sigma-Aldrich). Then, the sections were exposed to an avidin–biotin peroxidase complex for 2 h. The peroxidase activity was visualized using a stable diaminobenzidine solution. All immunoreactions were observed using a compound light microscope and these results were quantified using the ImageJ 1.46 (National Institutes of Health, Bethesda, MD, USA). For quantification, fragmentation (F) reflects that TH expression (brown patches in the image) that appeared as either single, homogeneous, and continuous or complex, heterogeneous, and discontinuous patches. Based on this protein expression classification, *F* was calculated as: *F* = *N*/*A*, where *N* is the number of protein expression patches in the image and *A* is the total area (in pixels) of the protein signal.

### 2.14 Molecular docking studies

Molecular docking studies were executed with Autodock4.2 software [60] using the solid-state nuclear magnetic resonance of α-syn fibrils retrieved from the RCSB Protein Data Bank (PDB code 2N0A). This structure holds 10 units of the peptide (140 amino acids), which are arranged in parallel by presenting features that commonly stabilize amyloid folds (such as intermolecular salt bridges, a glutamine ladder, steric zippers involving hydrophobic residues, and in-register parallel-β-sheet hydrogen-bonding with a Greek key motif) [61]. The embelin structure was downloaded from the PubChem database. Before performing docking, all water molecules were removed and polar hydrogen was added to α-syn fibrils by using the hydrogen module in AutoDock Tools, followed by assigning Kollman united atom partial charges. The grid maps representing the proteins in the actual docking process were calculated with AutoGrid. The grids were chosen to be sufficiently large to include the active site. The dimensions of the grids were thus 126 Å × 126 Å × 126 Å, with the spacing of 0.375 Å between the grid points. The *in silico* bonding mode of ligand with the receptor was carried out using the empirical free energy function and the Lamarckian genetic algorithm, applying the following standard protocol with an initial population of 150 randomly placed individuals, a maximum of 2.5 × 10^7^ energy evaluations, GA crossover mode = two points. A genetic algorithm of 100 independent docking cycles was carried out for the ligand. A genetic algorithm of 100 independent docking conformers was carried out for the compound, and the best conformer was taken based on their lowest binding energy for further analysis. PyMOL (https://pymol.org/2/) and LigPlot 2.2 tools were used to visualize the docking results [62]. The entire structure of embelin bound to the target protein was examined for binding energy and also for specific and nonspecific interacting residues.

### 2.15 Statistical analysis

The data are presented as the mean ± standard error of the mean. Differences among the groups were compared with a one-way analysis of variance (ANOVA), followed by the Bonferroni test for multiple comparisons. The *F* and *P* values for the one-way ANOVA are shown in each panel of each figure. Significant differences between groups are denoted by asterisks, with details indicated in each figure legend. SigmaPlot 14.5 (Systat, USA) was used for statistical analysis.

## 3 Results

### 3.1 Changes in brain MDA, NO, peroxynitrite, and urea levels

**Figure 1:**
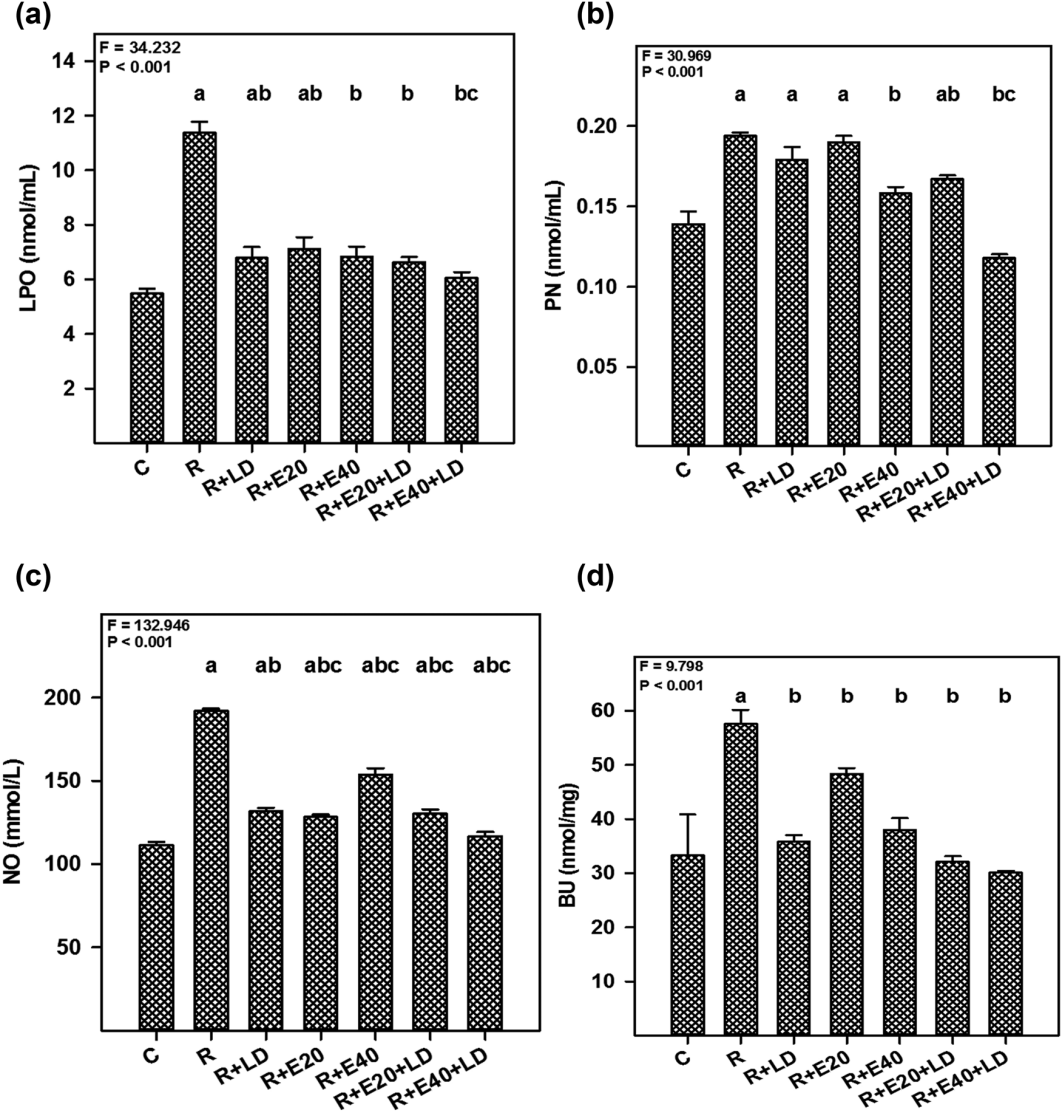
Antioxidant potential of embelin (E) and embelin plus LD therapies in mice treated with rotenone, based on the measurement of brain (a) LPO, denoted by MDA, (b) NO, (c) peroxynitrite (PN), and (d) brain urea (BU) levels. The values represent the mean ± standard error (*n* = 6). For the embelin groups, the number indicates the dose (in mg kg^−1^ body weight). The *F* and *P* values are based on the one-way analysis of variance. The level of significance was found to be *P* < 0.001. The superscript letters indicate the results of the Bonferroni multiple comparison test. ^a^Significantly different from the control group. ^b^Significantly different from the rotenone group. ^c^Significantly different from the rotenone + LD group.

The mean MDA level in rotenone-treated mice was more than two times higher than in the control mice (11.36 ± 0.40 and 5.48 ± 0.17 nmol mL^−1^, respectively; Figure 1a). The mice exposed to rotenone plus embelin and/or LD showed markedly reduced MDA compared with rotenone treatment alone (Figure 1a; *P* < 0.001). Notably, the combination of 40 mg kg^−1^ embelin and 7.5 mg kg^−1^ LD rendered even better protection against LPO. The mean peroxynitrite level in the rotenone-treated mice (group ІI) was markedly higher than the control mice (0.19 ± 0.001 and 0.13 ± 0.007 nmol mL^−1^, respectively, *P* < 0.001; Figure 1b). The addition of embelin and/or LD to rotenone-treated mice significantly reduced the peroxynitrite level compared with the rotenone-treated mice (*P* < 0.001). Rotenone-treated mice that received 40 mg kg^−1^ embelin or 40 mg kg^−1^ embelin plus 7.5 mg kg^−1^ LD showed almost similar peroxynitrite levels to the control mice (Figure 1b).

The mean NO level in rotenone-treated mice was significantly higher than in the control mice (19.0 ± 1.1 and 11.2 ± 1.9 µmol L^−1^, respectively, *P* < 0.001; Figure 1c). Administering embelin and/or LD to rotenone-treated mice markedly reduced NO levels compared with mice that only received rotenone (*P* < 0.001). In particular, the NO levels in the rotenone- and drug-treated mice (40 mg kg^−1^ embelin plus 7.5 mg kg^−1^ LD) were not different from the control group.

The mean brain urea in the rotenone-treated mice was nearly 75% higher than the control mice (57.60 ± 2.59 and 33.30 ± 7.51 nmol mg^−1^, respectively, *P* < 0.001; Figure 1d). The addition of embelin and/or LD to rotenone-treated mice markedly reduced the brain urea levels compared with mice that only received rotenone (*P* < 0.001). Indeed, treatment returned the urea level to near what was found in control mice. Based on the above findings, treatment with embelin alone or embelin plus LD reversed the biochemical alterations caused by rotenone administration.

### 3.2 Nurr1 protein changes in the SN

**Figure 2:**
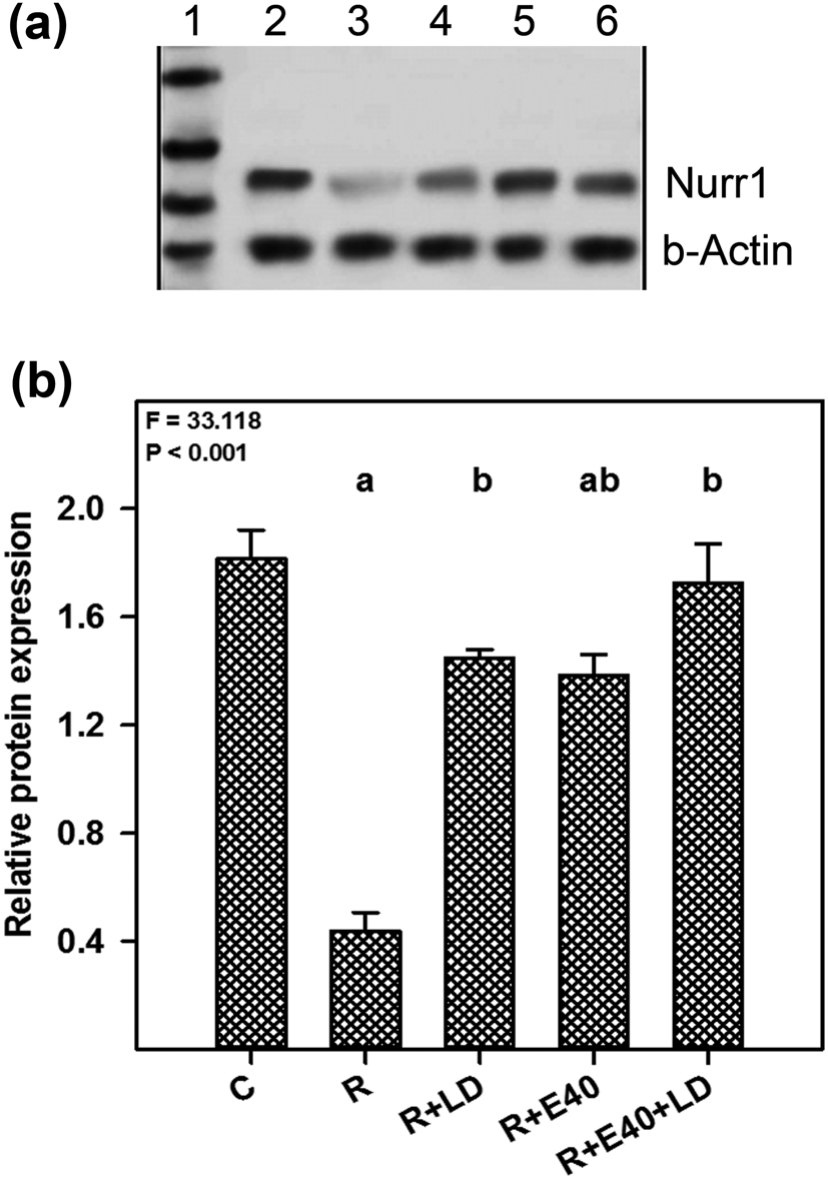
(a) Western blot analysis showing Nurr1 protein changes in the SN. Lane 1, molecular weight ladder; lane 2, control; lane 3, rotenone (R); lane 4, R + embelin (E, 40 mg kg^−1^); lane 5, R + E (40 mg kg^−1^) + LD (LD, 7.5 mg kg^−1^); lane 6, R + LD (7.5 mg kg^−1^). (b) The bar graph shows the relative expression of Nurr1 in various groups. The values present the mean ± standard error (*n* = 4). The *F* and *P* values are based on a one-way analysis of variance. The level of significance was found to be *P* < 0.001. The letters indicate the results of the Bonferroni multiple comparison test. ^a^Significantly different from the control group. ^b^Significantly different from the rotenone group. ^c^Significantly different from the rotenone + LD group.

Figure 2 shows the western blot analysis of Nurr1 (66 kDa) protein in the SN. Nurr1 expression in the rotenone-treated mice was significantly decreased (*P* < 0.05) compared with the control group. Nurr1 expression in the rotenone-treated mice administered LD standard therapy (7.5 mg kg^−1^) was restored to the control level. Embelin (40 mg kg^−1^) plus LD (7.5 mg kg^−1^) combination therapy in rotenone-treated mice also reverted the Nurr1 protein expression to almost the control level. However, the effect of embelin alone (40 mg kg^−1^) in restoring the Nurr1 protein level was comparatively less than the embelin plus LD combination therapy. Therefore, embelin plus LD combination therapy has a better protective effect on the brain dopaminergic system.

### 3.3 Gut histopathological changes and histomorphometry

**Figure 3:**
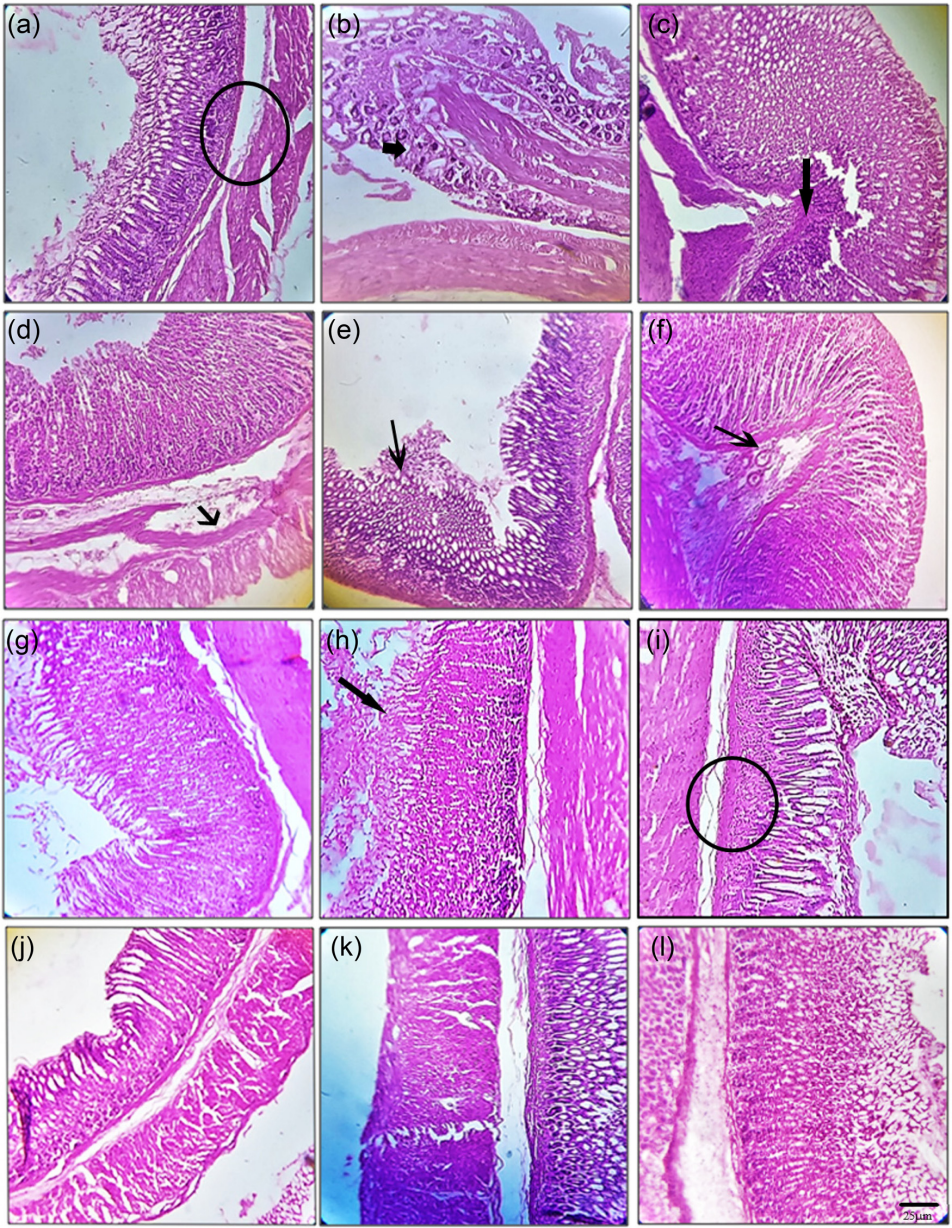
Representative micrographs of H&E staining of gut sections (*n* = 4). The control group (a) shows normal architecture of all three mucosal layers (circle), with normal crypts and no inflammatory cell infiltration. In the rotenone-treated group (c), there is destruction of the mucosa and surface epithelia (arrow mark). Moreover, inflammatory cell infiltration (small and thick arrow) is apparent in the lamina propria (b). In the rotenone + embelin (20 mg kg^−1^) group (d), there is mucosal edema (small and thin arrow), with intense inflammatory infiltration and crypt distortion (e). In the rotenone + embelin (40 mg kg^−1^) group, there were epithelial destruction and moderate inflammatory cell infiltration (f) apart from normal arrangement of all the three mucosal layers (g). In the rotenone + embelin (20 mg kg^−1^) + LD (7.5 mg kg^−1^) group, all three mucosal layers showed a normal arrangement (i, circle), although there were epithelial destruction and moderate inflammatory cell infiltration (h). In the rotenone + embelin (40 mg kg^−1^) + LD (7.5 mg kg^−1^) group, there were little damage, no edema, proper arrangement of mucosal layers, and limited inflammatory cell infiltration (j and k). The rotenone + LD (15 mg kg^−1^) group showed similar features (l). The scale bar is 25 µm.

Micrographs of H&E-stained gut sections are shown in Figure 3. The control mice showed normal architecture of all three mucosa levels (Figure 3a, circle), with normal crypts and no inflammatory cell infiltration. In the rotenone-treated mice (Figure 3b), there was remarkable destruction of the mucosa and surface epithelia (arrow mark). Moreover, inflammatory cell infiltration (arrow head) was noted in the lamina propria. In the group treated with 10 mg kg^−1^ embelin, there were epithelial destruction, mucosal edema (small arrow), intense inflammatory cell infiltration, and crypt distortion (Figure 3c), whereas in the group treated with 20 mg kg^−1^ embelin, there was inflammation in the mucosal layer but no damage to the epithelial layer (Figure 3d). Reduced crypt damage, less edema, and less inflammation were seen in this group. In the group treated with 10 mg kg^−1^ embelin plus LD (Figure 3e), there were epithelial destruction and moderate infiltration; normal arrangement of all the three mucosal layers is visible. However, in the groups treated with 20 mg kg^−1^ embelin plus LD (Figure 3f) or LD alone (Figure 3g), there were less damage in gut tissues, no edema, proper arrangement of mucosal layers, and less inflammatory cell infiltration.

Based on a scoring system established by Okayasu et al. [63], inflammatory cell infiltrates, epithelial changes, and the mucosal architecture were scored based on specific criteria. The changes in the gut histomorphometry were analyzed and the overall scores for each group are presented in Table 1. Similar to the histopathological changes, gut from mice treated with 20 mg kg^−1^ embelin plus LD showed substantial improvement compared with the other treated groups.

**Table 1:** Scoring pattern for gut histomorphometry

Number	Groups	Histomorphometry	Mean score
1	C	Intact surface epithelium without inflammatory cells	0
2	R	Infiltration of inflammatory cells, mucosal ulceration, irregular crypts, hyperplasia, goblet cell loss, cryptitis, and erosion	3.8
3	R + LD	Infiltration of inflammatory cells, hyperplasia, goblet cell loss, cryptitis, and erosion	2.6
4	R + E20	Infiltration of inflammatory cells extended to the submucosa, hyperplasia, marked epithelial changes, goblet cell loss, irregular crypts, and erosion	3.7
5	R + E40	Infiltration of inflammatory cells, goblet cell loss, crypt loss, and erosion	2.5
6	R + E20 + LD	Infiltration of inflammatory cells, goblet cell loss, crypt loss, and erosion	1.7
7	R + E40 + LD	Minimal infiltration of inflammatory cells, goblet cell loss and crypt loss with granular tissue repair, and new capillary formation	1.0

C, control; R, rotenone; LD, levodopa; E, embelin (20 or 40 mg kg^−1^).

Scoring – no effect = 0; minimal = 1; mild = 2; moderate = 3; severe = 4.

The mean score is an average of three animals in each group.

### 3.4 Effect of embelin and embelin plus LD treatments on the rotenone-induced reduction of the TH-positive cells in the SN

**Figure 4:**
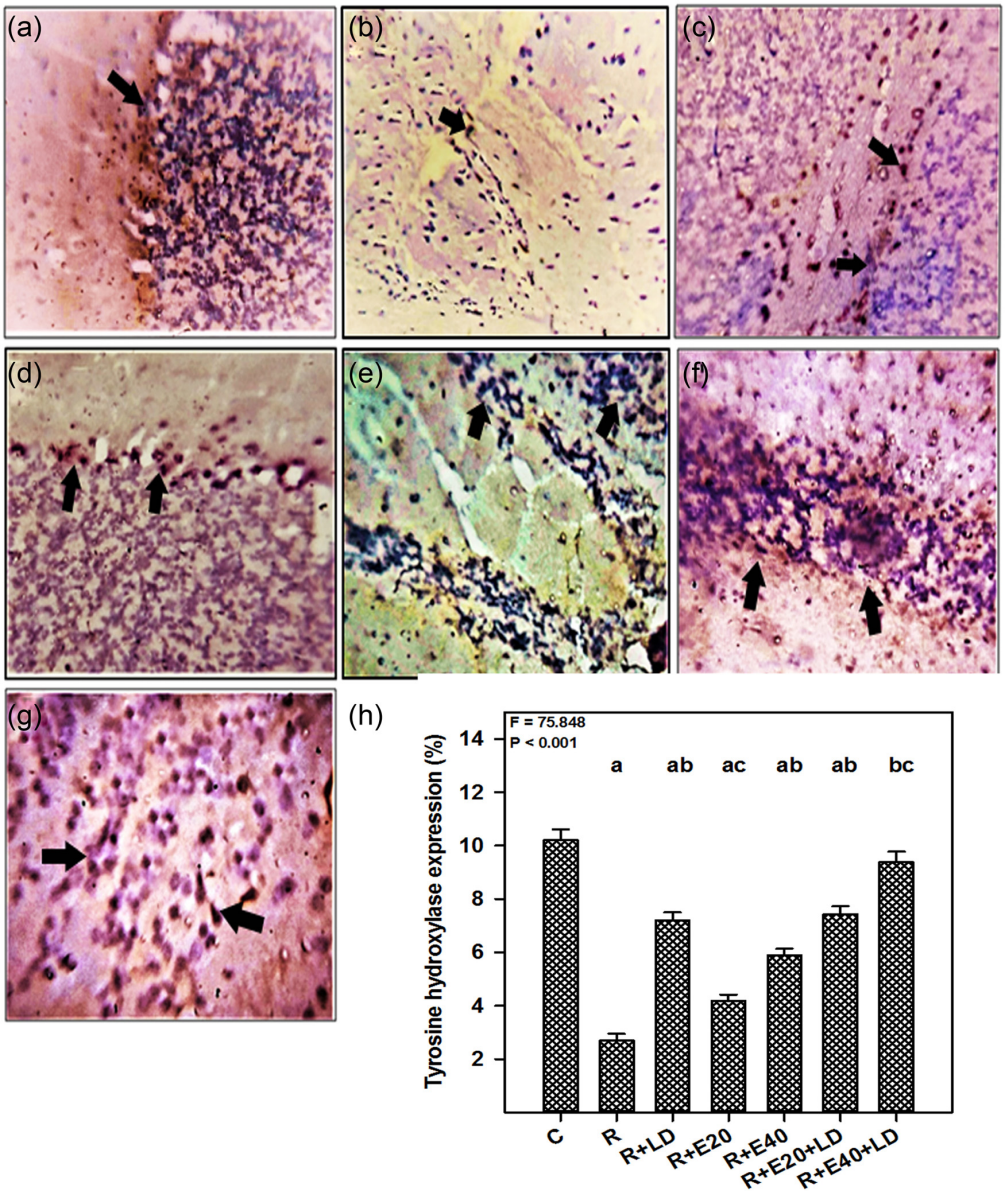
The effect of embelin and embelin + LD therapies on the TH protein expression level. TH immunostaining in the SN from the (a) control, (b) rotenone, (c) rotenone + embelin (20 mg kg^−1^), (d) rotenone + embelin (40 mg kg^−1^), (e) rotenone + embelin (20 mg kg^−1^) + LD (7.5 mg kg^−1^), (f) rotenone + embelin 40 mg kg^−1^ + LD 7.5 mg kg^−1^, and (g) rotenone + LD (7.5 mg kg^−1^) groups. The arrows denote the TH-positive cells. (h) Quantitative analysis of TH protein expression. The results are expressed as the mean + standard error of the mean (*n* = 4). The *F* and *P* values are based on the one-way analysis of variance with Bonferroni multiple comparison test. The level of significance was found to be *P* < 0.001. ^a^Significantly different from the control group. ^b^Significantly different from the rotenone group. ^c^Significantly different from the rotenone + LD group.

TH catalyzes the rate-limiting step in DA biosynthesis in dopaminergic neurons. The number of TH-positive cells was evaluated in the SN (Figure 4). Compared with the control group (Figure 4a), there was a marked reduction in TH-positive cells in the rotenone-treated mice (Figure 4b). By contrast, in the drug-treated groups (Figure 4c–g), there was protection of TH-positive cells. Figure 4h shows the percentage of TH-positive cells.

### 3.5 *In silico* studies revealed the binding affinity of embelin for α-syn fibrils

**Figure 5:**
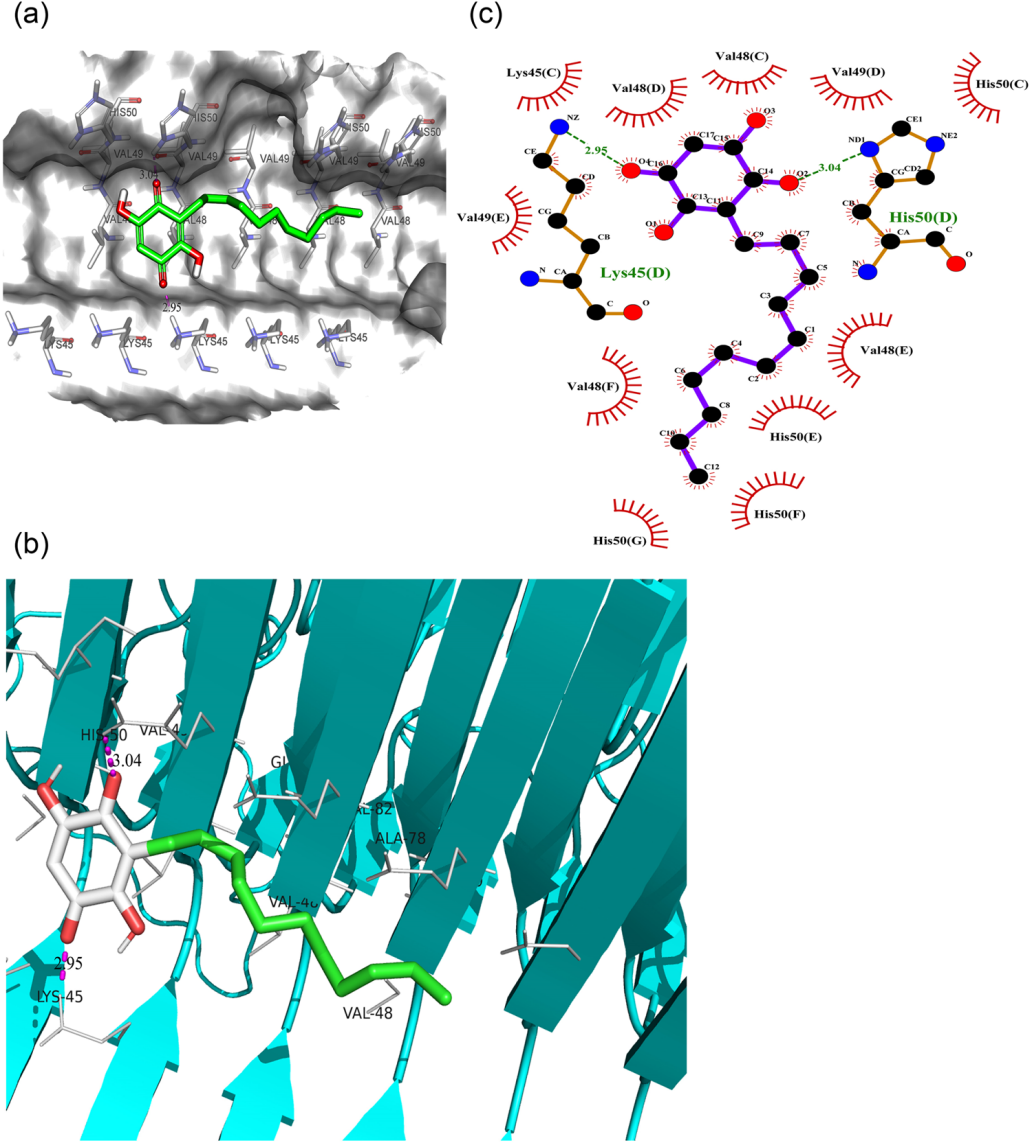
(a) An illustration of embelin (green structure) in the substrate-binding pocket of α-syn. The crucial residues of α-syn are shown as atom stick figures. (b) The secondary structure view of the embelin–α-syn complex. Hydrogen bonds are represented by the magenta dashed line. (c) A LigPlot view of the embelin–α-syn complex. The Lys45(D) and His50(D) residues of α-syn are highlighted. The image shows the hydrogen bonds and hydrophobic and van der Waals interactions that mediate embelin–α-syn binding.

The binding mode of embelin with α-syn fibrils was analyzed using the Autodock 4.2 suite. Thirteen putative binding sites were identified (Figure 5a). Site 2 (with residues Y39, S42, and T44), site 9 (with residues G86, F94, and K96), and site 3/13 (with residues L43, L45, V48, and H50) showed a high probability of interaction [64]. The embelin scoring energy (−4.18 kcal mol^−1^) is most favorable for interaction with site 3/13. For this interaction, two hydrogen bonds are formed. The first hydrogen bond is between the second oxygen atom of embelin with the ND1 group of the polar residue His50(D), with a bond distance of 3.0 Å (Figure 5b and c). The second hydrogen bond is between the fourth oxygen atom of embelin and the NZ group of positively charged residue Lys45(D), with a bond distance of 2.9 Å (Figure 5b and c). In addition, residues Val48(C), Val48(D), Val48(F), His50(G), and His50(F) are involved in hydrophobic interactions to sustain the stability of the embelin–α-syn complex. Furthermore, van der Waals interactions occur with the residues Lys45(C), Val48(E), Val49(C), Val49(D), Val49(E), Val49(F), His50(C), and His50(E) (Figure 5b and c).

## 4 Discussion

Chronic intraperitoneal administration of rotenone induces a gradual movement disorder that recapitulates PD symptoms [54]. The rotenone-treated mice in this study developed similar symptoms, and the herbal drug embelin ameliorated oxidative stress and associated complications in these mice. Importantly, embelin plus LD therapy rendered better protective effects against PD-related dysfunction compared with embelin treatment alone. Of several molecular mechanisms proposed in the etiology and pathogenesis of PD, oxidative stress and ROS generation play key roles in certain regions of the brain of people with PD by triggering mitochondrial dysfunction [65,66,67]. The embelin treatment doses in the present study were slightly different from earlier works [68,69]; they were chosen by taking into consideration the ability of embelin to protect the brain against oxidative stress and inflammation. In the present work, although a 50% reduced dose of LD (7.5 mg kg^−1^) was administered to the rotenone-treated mice [70], a previous study supported this dose as a therapy for an animal model of PD [71].

After entering neurons, rotenone blocks mitochondrial complex I activity, increases ROS production, and inhibits proteasome activity, generating proteolytic stress. Rotenone decreases DA and reduced glutathione (GSH) levels and increases LPO in dopaminergic neurons, resulting in oxidative damage [72,73]. The significantly elevated levels of brain lipid peroxides, NO, peroxynitrite, and urea in the rotenone-treated mice suggest the effect of rotenone treatment on oxidative stress-related damage, implicating free radicals and defective mitochondrial respiration [24,74,75,76]. Therefore, increased generation of reactive oxygen metabolites and the ensuing oxidative stress are considered important pathogenic mechanisms that underlie rotenone-induced cell death. By contrast, embelin and embelin plus LD combination therapies in rotenone-treated mice exerted an antioxidative effect by reversing the changes in brain levels of LPO, peroxynitrite, NO, and urea. These effects might have contributed to the neuroprotective and therapeutic benefits exerted by the compounds. Hence, the antioxidant and neuroprotective properties of embelin [44,45,46,77,78,79] and the ability of LD to improve the brain DA availability and nerve conduction should have ameliorated the rotenone-induced symptoms in the mice.

Increased NO generation is also an important contributor to the mechanisms by which rotenone mediates neuronal damage [80]. Rotenone increases the expression of inducible nitric oxide synthase (iNOS) in the striatum and SN and increases the level of NO in rodent brains [81]. Increased NO generation via neuronal nitric oxide synthase could also be involved in rotenone-mediated neurotoxicity. This increase in the NOS activity and 3-nitrotyrosine and nigrostriatal damage in rats treated with rotenone was reduced by administration of the neuronal NOS inhibitor 7-nitroindazole [80]. Evidence implicates increased NO formation for a prolonged time during inflammation and toxicity as a cause of neuronal death. The reaction of NO with molecular oxygen yields reactive oxides of nitrogen, including nitrogen dioxide (NO_2_) and dinitrogen trioxide (N_2_O_3_). The reaction of NO with the superoxide anion ( {\text{O}}_{2}^{\cdot -}] ) forms reactive peroxynitrite; this species leads to oxidation, nitrosylation of thiols in proteins or GSH, and nitration of tyrosine residues in proteins [82,83]. Oxidative/nitrosative stress and inactivation of several mitochondrial electron transport complexes, including complex I, cause mitochondrial dysfunction, inhibition of mitochondrial respiration, depletion of cellular energy, and, ultimately, neuronal cell death [84]. In agreement with previous reports [45,46,77], the neuroprotective effect of embelin against PD observed in the present study could be attributed to its antioxidant and free radical scavenging capacity. The amelioration of brain urea levels in PD brains by embelin and embelin plus LD combination therapies implicates their intricate role in the restoration of brain energy functions. Notably, treatment with 40 mg kg^−1^ embelin or 40 mg kg^−1^ embelin plus LD provided the best effects.

The nuclear receptor transcription factor Nurr1, through its interaction with several key factors, is crucial for mesencephalic dopaminergic neuron survival. Specifically, this transcription factor contributes to regulate the expression of TH, DA transporter, vesicular monoamine transporter 2, and aromatic l-amino acid decarboxylase and to support the production and storage of DA, a critical brain neurotransmitter [85]. Reduced Nurr1 expression that is observed in PD might not only result in dopaminergic neuron loss and motor impairments but also impair mitochondrial function and oxidative phosphorylation [86]. Consistent with the fact that Nurr1 is vital for the development and survival of midbrain dopaminergic neurons, studies have reported that overexpression of α-syn (WT or an A53T mutant) in mice caused a striking reduction in Nurr1 protein, inhibited nuclear factor kappa B (NF-κB) expression and transcriptional activity, and decreased binding of NF-κB to the Nurr1 promoter [86]. Moreover, Nurr1 expression in microglia and astrocytes might help protect dopaminergic neurons from inflammation-associated death. Therefore, the anti-inflammatory role of Nurr1 seems important in the suppression of PD pathology [87,88]. Because Nurr1 helps regulate dopaminergic neuron functions via neurotrophic signaling by the glial-derived growth factor (GDNF) receptor Ret, the GDNF–Ret–Nurr1 pathway may play a major role in protecting dopaminergic neurons and their functions. Moreover, interaction between Nurr1 and Foxa2 is critical for the protection of midbrain dopaminergic neurons from toxic insults [89]. As a regulator of neuroprotective genes, Nurr1 drives the cAMP response element-binding protein-mediated neuroprotective response in neurons following oxidative stress and excitotoxic insults [90].

While LD has provided great benefit to patients with PD, its chronic use is associated with issues. For example, patients can develop non-motor behavioral disorders, including impulse control disorders, psychosis, hallucinations, and (hypo)mania [91]. Moreover, chronic LD treatment can lead to oxidative stress [92,93]. The ability to add a natural compound could allow reducing the LD dose administered to patients while preventing the negative consequences that could results from this dose reduction. This potential requires additional studies, including the examination of behavioral changes in animal models of PD.

The survival of dopaminergic neurons relies on two key proteins, namely HIF-1α and Nurr1. Therefore, researchers have proposed that PD pathogenesis could be due to the deficiencies in these genes [30]. Therapeutic agents that increase the expression of these two genes ameliorated PD by restoring striatal DA content and motor functions and mitigated the PD-induced histological changes in SN [30]. As embelin and the embelin plus LD combination considerably increased the expression of Nurr1 protein in the midbrain region of rotenone-treated mice, the findings collectively indicate that Nurr1 could be a promising therapeutic target [86] by which degenerating midbrain dopaminergic neurons could be protected in a bidirectional mode by promoting intracellular survival pathways in midbrain dopaminergic neurons as well as by modulating the toxic environment surrounding these neurons. The binding affinity of embelin toward the active site of α-syn was predicted by *in silico* molecular docking. The findings emphasize the ability of embelin to bind to and dissolve α-syn fibrils. These fibrils are 1,000-fold more toxic than the oligomers or other precursors with regard to motor impairment, dopaminergic neuronal loss, and synaptic damage [94,95]. Therefore, the docking results in this study implicate that embelin can likely bind to α-syn fibrils and break them up, an eventuality that could protect the organism from α-syn-mediated toxicity. Taken altogether, embelin in combination with LD could be a viable treatment for PD.

Several works have substantiated gut pathology and damage in patients with PD as well as PD animal models. Some authors have speculated that PD originates outside of the CNS through some involvement with the ENS [96,97]. Indeed, Lewy bodies are found at every layer of the GI tract in patients with PD [98]. In support of this hypothesis, several studies have demonstrated that the gut microbiota may contribute to PD [99,100,101], and these effects could increase α-syn expression in the gut. While there is growing evidence that emphasizes the importance of both brain and gut pathological changes in PD, there is no definitive proof as to which organ is affected first. The gut histopathological alterations caused by systemic rotenone administration suggest colonic inflammation [100]. Hence, intestinal dysfunction-related problems could be involved in PD progression [102]. Moreover, the increase in the cellularity of the lamina propria in the colon provides evidence for the occurrence of irritable bowel syndrome that affects the colonic mucosa [103]. There is plausible proof of a link between gut bacteria and neurodegenerative diseases [104,105] that affects the ENS. Thus, the gut microbiota is implicated in motor dysfunction, neuronal inflammation, and α-syn pathology.

Several studies indicate that patients with inflammatory bowel disease, which is known to increase intestinal permeability, have an increased risk of developing PD. This eventuality suggests a role for GI inflammation in the development of PD [106]. PD is also closely associated with inflammation caused by specific microbial cell structures and pattern recognition receptor signaling pathways. Inflammation in the ENS could be due to increased accumulation of *Escherichia coli* [93]. Evidence supports that gut dysfunction might aggravate oxidative stress and mucosal inflammation in PD apart from inducing α-syn accumulation in the ENS [107]. Both inflammation and oxidative stress play a synergistic key role in gut dysbiosis-mediated changes in gut DA production as well as secretion of DA in the SN through activation of microglia NLRP3 inflammasome and iNOS expression [108]. The restoration of gut damage in rotenone-treated mice by embelin and embelin plus LD therapies implicates the antimicrobial, [109], antioxidant [76,110,111], and anti-inflammatory functions of embelin [78,112,113] and the antioxidant capacity [114,115] and gut epithelium protection rendered by LD [116]. Overall, our earlier investigation [53] and the present findings have substantiated embelin’s therapeutic potential.

We used the well-established rotenone model of PD, which was first reported in 2000 [117]. Since this report, there has been a debate as to how well this model recapitulates the PD phenotype. There have been efforts to improve the administration of rotenone to achieve a consistent phenotype, and it serves as useful model to test neuroprotective strategies [118]. Moreover, mice treated with rotenone develop two of the hallmarks of PD: degradation of dopaminergic neurons in the SN and Lewy body formation in the surviving dopaminergic neurons [119]. Another issue is that rotenone induces an acute lesion – dopaminergic cell death and DA deficiency – whereas PD is a chronic, degenerative disease. However, we still feel that the rotenone model is appropriate to test potentially neuroprotective compounds. Our findings underscore the benefit of this compound to ameliorate hallmarks of PD, and this compound could be tested in other models of PD, including those generated by altering the genes that have been implicated in familial PD [120]. Like embelin, other natural products have also proven their efficacies when tested in both *in vitro* and *in vivo* models of PD [121,122], thereby implicating the importance of herbal treatment in PD.

## 5 Conclusion

The findings from this study demonstrate that embelin could be a potential drug for PD treatment, either alone or in combination with LD. Embelin attenuates rotenone toxicity to protect the brain through antioxidant mechanisms as well as by the upregulation of Nurr1 and TH proteins. Notably, *in silico* binding studies revealed that embelin can bind to α-syn fibrils and break up these fibrils. This could ameliorate the deficits caused by α-syn aggregation. Overall, embelin is a promising molecule for the treatment of PD.

## References

[1] Myhre O, Utkilen H, Duale N, Brunborg G, Hofer T. Metal dyshomeostasis and inflammation in Alzheimer’s and Parkinson’s diseases: possible impact of environmental exposures. Oxid Med Cell Longev. 2013;2013:1–19. https://www.hindawi.com/journals/omcl/2013/726954/10.1155/2013/726954 PMC3654362 23710288

[2] Emamzadeh FN, Surguchov A. Parkinson’s disease: biomarkers, treatment, and risk factors. Front Neurosci. 2018;12:1–14. 10.3389/fnins.2018.00612. PMC6125353 30214392

[3] Shulman LM, Taback RL, Bean J, Weiner WJ. Comorbidity of the nonmotor symptoms of Parkinson’s disease. Mov Disord. 2001;16(3):507–10. 10.1002/mds.1099 11391746

[4] Tan JM, Wong ES, Lim KL. Protein misfolding and aggregation in Parkinson’s disease. Antioxid Redox Signal. 2009;11(9):2119–34. 10.1089/ars.2009.2490 19243238

[5] Blesa J, Trigo-Damas I, Quiroga-Varela A, Jackson-Lewis VR. Oxidative stress and Parkinson’s disease. Front Neuroanat. 2015;9:1–9. 10.3389/fnana.2015.00091. PMC4495335 26217195

[6] Michel PP, Hirsch EC, Hunot S. Understanding dopaminergic cell death pathways in Parkinson disease. Neuron. 2016;90(4):675–91. 10.1016/j.neuron.2016.03.038 27196972

[7] Hemmati-Dinarvand M, Saedi S, Valilo M, Kalantary-Charvadeh A, Sani MA, Kargar R, et al. Oxidative stress and Parkinson’s disease: conflict of oxidant-antioxidant systems. Neurosci Lett. 2019;709:134296. 10.1016/j.neulet.2019.134296 31153970

[8] Lynch-Day MA, Mao K, Wang K, Zhao M, Klionsky DJ. The role of autophagy in Parkinson’s disease. Cold Spring Harb Perspect Med. 2012;2(4):a009357. 10.1101/cshperspect.a009357 PMC3312403 22474616

[9] Bové J, Prou D, Perier C, Przedborski S. Toxin-induced models of Parkinson’s disease. NeuroRx. 2005;2(3):484–94. 10.1602/neurorx.2.3.484 PMC1144492 16389312

[10] Cannon JR, Greenamyre JT. The role of environmental exposures in neurodegeneration and neurodegenerative diseases. Toxicol Sci. 2011;124(2):225–50. 10.1093/toxsci/kfr239 PMC3216414 21914720

[11] Brown TP, Rumsby PC, Capleton AC, Rushton L, Levy LS. Pesticides and Parkinson’s disease – is there a link? Environ Health Perspect. 2006;114(2):156–64. 10.1289/ehp.8095 PMC1367825 16451848

[12] Cicchetti F, Drouin-Ouellet J, Gross RE. Environmental toxins and Parkinson’s disease: what have we learned from pesticide-induced animal models? Trends Pharmacol Sci. 2009;30(9):475–83. 10.1016/j.tips.2009.06.005 19729209

[13] Abdulwahid Arif I, Ahmad Khan H. Environmental toxins and Parkinson’s disease: putative roles of impaired electron transport chain and oxidative stress. Toxicol Ind Health. 2010;26(2):121–8. 10.1177/0748233710362382 20207656

[14] Nandipati S, Litvan I. Environmental Exposures and Parkinson’s Disease. Int J Environ Res Public Health. 2016:1–19. https://www.mdpi.com/1660-4601/13/9/881 10.3390/ijerph13090881 PMC5036714 27598189

[15] Parker WD Jr, Boyson SJ, Parks JK. Abnormalities of the electron transport chain in idiopathic Parkinson’s disease. Ann Neurol. 1989;26(6):719–23. 10.1002/ana.410260606 2557792

[16] Schapira AH, Cooper JM, Dexter D, Clark JB, Jenner P, Marsden CD. Mitochondrial complex I deficiency in Parkinson’s disease. J Neurochem. 1990;54(3):823–7. 10.1111/j.1471-4159.1990.tb02325.x 2154550

[17] Bindoff LA, Birch-Machin MA, Cartlidge NE, Parker WD Jr, Turnbull DM. Respiratory chain abnormalities in skeletal muscle from patients with Parkinson’s disease. J Neurol Sci. 1991;104(2):203–8. 10.1016/0022-510x(91)90311-t 1658241

[18] Keeney PM, Xie J, Capaldi RA, Bennett JP Jr. Parkinson’s disease brain mitochondrial complex I has oxidatively damaged subunits and is functionally impaired and misassembled. J Neurosci. 2006;26(19):5256–64. 10.1523/JNEUROSCI.0984-06.2006 PMC6674236 16687518

[19] Mounsey RB, Teismann P. Mitochondrial dysfunction in Parkinson’s disease: pathogenesis and neuroprotection. Parkinsons Dis. 2010;2011;1–18. 10.4061/2011/617472. PMC3014704 21234411

[20] Hansmannel F, Sillaire A, Kamboh MI, Lendon C, Pasquier F, Hannequin D, et al. Is the urea cycle involved in Alzheimer’s disease? J Alzheimers Dis. 2010;21(3):1013–21. 10.3233/JAD-2010-100630 PMC2945690 20693631

[21] Handley RR, Reid SJ, Brauning R, Maclean P, Mears ER, Fourie I, et al. Brain urea increase is an early Huntington’s disease pathogenic event observed in a prodromal transgenic sheep model and HD cases. Proc Natl Acad Sci USA. 2017;114(52):E11293–302. 10.1073/pnas.1711243115 PMC5748180 29229845

[22] Sivanesan S, Mundugaru R, Rajadas J. Possible clues for brain energy translation via endolysosomal trafficking of APP-CTFs in Alzheimer’s disease. Oxid Med Cell Longev. 2018;2018:1–11. 10.1155/2018/2764831. PMC6215552 30420907

[23] Sivanesan S, Chang E, Howell MD, Rajadas J. Amyloid protein aggregates: new clients for mitochondrial energy production in the brain? FEBS J. 2020;287(16):3386–95. 10.1111/febs.15225 31981301

[24] Kavuri S, Sivanesan S, Howell MD, Vijayaraghavan R, Rajadas J. Studies on Parkinson’s-disease-linked genes, brain urea levels and histopathology in rotenone induced Parkinson’s disease rat model. World J Neurosci. 2020;10(4):216–34.

[25] Le W, Pan T, Huang M, Xu P, Xie W, Zhu W, et al. Decreased NURR1 gene expression in patients with Parkinson’s disease. J Neurol Sci. 2008;273(1–2):29–33. 10.1016/j.jns.2008.06.007 PMC2572302 18684475

[26] Kadkhodaei B, Ito T, Joodmardi E, Mattsson B, Rouillard C, Carta M. Nurr1 is required for maintenance of maturing and adult midbrain dopamine neurons. J Neurosci. 2009;29(50):15923–32. 10.1523/JNEUROSCI.3910-09.2009 PMC6666174 20016108

[27] Dong J, Li S, Mo JL, Cai HB, Le WD. Nurr1-based therapies for Parkinson’s disease. CNS Neurosci Ther. 2016;22(5):351–9. 10.1111/cns.12536 PMC4833611 27012974

[28] Chu Y, Kompoliti K, Cochran EJ, Mufson EJ, Kordower JH. Age-related decreases in Nurr1 immunoreactivity in the human substantia nigra. J Comp Neurol. 2002;450(3):203–14. 10.1002/cne.10261 12209851

[29] Le W, Pan T, Huang M, Xu P, Xie W, Zhu W, et al. Decreased NURR1 gene expression in patients with Parkinson’s disease. J Neurol Sci. 2008;273(1-2):29–33. 10.1016/j.jns.2008.06.007 PMC2572302 18684475

[30] Kandil EA, Sayed RH, Ahmed LA, Abd El Fattah MA, El-Sayeh BM. Hypoxia-inducible factor 1 alpha and nuclear-related receptor 1 as targets for neuroprotection by albendazole in a rat rotenone model of Parkinson’s disease. Clin Exp Pharmacol Physiol. 2019;46(12):1141–50. 10.1111/1440-1681.13162 31408200

[31] Paliga D, Raudzus F, Leppla SH, Heumann R, Neumann S. Lethal factor domain-mediated delivery of Nurr1 transcription factor enhances tyrosine hydroxylase activity and protects from neurotoxin-induced degeneration of dopaminergic cells. Mol Neurobiol. 2019;56(5):3393–403. 10.1007/s12035-018-1311-6 PMC6476859 30121937

[32] Avetisyan M, Schill EM, Heuckeroth RO. Building a second brain in the bowel. J Clin Invest. 2015;125(3):899–907. 10.1172/JCI76307 PMC4362233 25664848

[33] Santos SF, de Oliveira HL, Yamada ES, Neves BC, Pereira A Jr. The gut and Parkinson’s disease-a bidirectional pathway. Front Neurol. 2019;10:1–8. 10.3389/fneur.2019.00574. PMC6558190 31214110

[34] Cryan JF, Dinan TG. Mind-altering microorganisms: the impact of the gut microbiota on brain and behaviour. Nat Rev Neurosci. 2012;13(10):701–12. 10.1038/nrn3346 22968153

[35] Clairembault T, Leclair-Visonneau L, Coron E, Bourreille A, Le Dily S, Vavasseur F, et al. Structural alterations of the intestinal epithelial barrier in Parkinson’s disease. Acta Neuropathol Commun. 2015;3:1–9. 10.1186/s40478-015-0196-0. PMC4353469 25775153

[36] Klingelhoefer L, Reichmann H. Pathogenesis of Parkinson disease – the gut-brain axis and environmental factors. Nat Rev Neurol. 2015;11(11):625–36. 10.1038/nrneurol.2015.197 26503923

[37] Yang D, Zhao D, Ali Shah SZ, Wu W, Lai M, Zhang X, et al. The role of the gut microbiota in the pathogenesis of Parkinson’s disease. Front Neurol. 2019;10:1–13. 10.3389/fneur.2019.01155. PMC6851172 31781020

[38] Dick FD, De Palma G, Ahmadi A, Scott NW, Prescott GJ, Bennett J, et al. Environmental risk factors for Parkinson’s disease and parkinsonism: the Geoparkinson study. Occup Environ Med. 2007;64(10):666–72. 10.1136/oem.2006.027003 PMC2078401 17332139

[39] Weisskopf MG, Weuve J, Nie H, Saint-Hilaire MH, Sudarsky L, Simon DK, et al. Association of cumulative lead exposure with Parkinson’s disease. Environ Health Perspect. 2010;118(11):1609–13. 10.1289/ehp.1002339 PMC2974701 20807691

[40] Cicchetti F, Drouin-Ouellet J, Gross RE. Environmental toxins and Parkinson’s disease: what have we learned from pesticide-induced animal models? Trends Pharmacol Sci. 2009;30(9):475–83. 10.1016/j.tips.2009.06.005 19729209

[41] Jiang T, Sun Q, Chen S. Oxidative stress: a major pathogenesis and potential therapeutic target of antioxidative agents in Parkinson’s disease and Alzheimer’s disease. Prog Neurobiol. 2016;147:1–19. 10.1016/j.pneurobio.2016.07.005 27769868

[42] Rybakowska I, Szreder G, Kaletha K, Barwina M, Waldman W, Sein Anand J. Reactive oxygen species and 3,4-dihydroxyphenylacetaldehyde in pathogenesis of Parkinson disease. Przegl Lek. 2011;68(8):486–7. 22010445

[43] Calixto JB. The role of natural products in modern drug discovery. An Acad Bras Cienc. 2019;91(S3):1–7. 10.1590/0001-3765201920190105. 31166478

[44] Kumar S, Deshmukh R. Embelin as a potential drug molecule: a review. J Pharmacogn Nat Prod. 2017;3:1–7. 10.4172/2472-0992.1000144.

[45] Bhuvanendran S, Kumari Y, Othman I, Shaikh MF. Amelioration of cognitive deficit by embelin in a scopolamine-induced Alzheimer’s disease-like condition in a rat model. Front Pharmacol. 2018;9:1–12. 10.3389/fphar.2018.00665. PMC6026638 29988493

[46] Dhadde SB, Nagakannan P, Roopesh M, Kumar SA, Thippeswamy BS, Veerapur VP, et al. Effect of embelin against 3-nitropropionic acid-induced Huntington’s disease in rats. Biomed Pharmacother. 2016;77:52–8. 10.1016/j.biopha.2015.11.009 26796265

[47] Pathan SA, Iqbal Z, Zaidi S, Talegaonkar S, Vohra D, Jain GK. CNS drug delivery systems: novel approaches. Recent Pat Drug Deliv Formul. 2009;3(1):71–89. 10.2174/187221109787158355 19149731

[48] Poojari R, Gupta S, Maru G, Khade B, Bhagwat S. Chemopreventive and hepatoprotective effects of embelin on N-nitrosodiethylamine and carbon tetrachloride induced preneoplasia and toxicity in rat liver. Asian Pac J Cancer Prev. 2010;11(4):1015–20. 21133617

[49] Edderkaoui M, Lugea A, Hui H, Eibl G, Lu QY, Moro A, et al. Ellagic acid and embelin affect key cellular components of pancreatic adenocarcinoma, cancer, and stellate cells. Nutr Cancer. 2013;65(8):1232–44. 10.1080/01635581.2013.832779 PMC3909533 24127740

[50] Thanvi BR, Lo TC. Long term motor complications of levodopa: clinical features, mechanisms, and management strategies. Postgrad Med J. 2004;80(946):452–8. 10.1136/pgmj.2003.013912 PMC1743071 15299154

[51] Encarnacion EV, Hauser RA. Levodopa-induced dyskinesias in Parkinson’s disease: etiology, impact on quality of life, and treatments. Eur Neurol. 2008;60(2):57–66. 10.1159/000131893 18480609

[52] Chitra M, Sukumar E, Suja V, Devi CS. Antitumor, anti-inflammatory and analgesic property of embelin, a plant product. Chemotherapy. 1994;40(2):109–3. 10.1159/000239181 7510605

[53] Koppal A, Sivanesan S, Vagdevi HR, Ethirajan S, Vijayaraghavan R. Embelin and levodopa combination therapy mitigates Parkinson’s disease complications in mice. Ind J Pharm Educ Res. 2021;55(2s):s468–78.

[54] Alam M, Schmidt WJ. Rotenone destroys dopaminergic neurons and induces parkinsonian symptoms in rats. Behav Brain Res. 2002;136(1):317–24. 10.1016/s0166-4328(02)00180-8 12385818

[55] Alam M, Schmidt WJ. L-DOPA reverses the hypokinetic behaviour and rigidity in rotenonetreated rats. Behav Brain Res. 2004;153:439–46. 10.1016/j.bbr.2003.12.021 15265640

[56] Ohkawa H, Ohishi N, Yagi K. Assay for lipid peroxides in animal tissues by thiobarbituric acid reaction. Anal Biochem. 1979;95(2):351–8. 10.1016/0003-2697(79)90738-3 36810

[57] Jabłońska E, Kiersnowska-Rogowska B, Ratajczak W, Rogowski F, Sawicka-Powierza J. Reactive oxygen and nitrogen species in the course of B-CLL. Adv Med Sci. 2007;52:154–8. 18217409

[58] Beckman JS, Ischiropoulos H, Zhu L, van der Woerd M, Smith C, Chen J. Kinetics of superoxide dismutase- and iron-catalyzed nitration of phenolics by peroxynitrite. Arch Biochem Biophys. 1992;298(2):438–45. 10.1016/0003-9861(92)90432-v 1416975

[59] Patassini S, Begley P, Reid SJ, Xu J, Church SJ, Curtis M. Identification of elevated urea as a severe, ubiquitous metabolic defect in the brain of patients with Huntington’s disease. Biochem Biophys Res Commun. 2015;468(1–2):161–6. 10.1016/j.bbrc.2015.10.140 26522227

[60] Morris GM, Huey R, Lindstrom W, Sanner MF, Belew RK, Goodsell DS, et al. AutoDock4 and AutoDockTools4: automated docking with selective receptor flexibility. J Comput Chem. 2009;30(16):2785–91. 10.1002/jcc.21256 PMC2760638 19399780

[61] Tuttle MD, Comellas G, Nieuwkoop AJ, Covell DJ, Berthold DA, Kloepper KD, et al. Solid-state NMR structure of a pathogenic fibril of full-length human α-synuclein. Nat Struct Mol Biol. 2016;23(5):409–15. 10.1038/nsmb.3194 PMC5034296 27018801

[62] Laskowski RA, Swindells MB. LigPlot+: multiple ligand-protein interaction diagrams for drug discovery. J Chem Inf Model. 2011;51(10):2778–86. 10.1021/ci200227u 21919503

[63] Okayasu I, Hatakeyama S, Yamada M, Ohkusa T, Inagaki Y, Nakaya R. A novel method in the induction of reliable experimental acute and chronic ulcerative colitis in mice. Gastroenterology. 1990;98(3):694–702. 10.1016/0016-5085(90)90290-h 1688816

[64] Hsieh CJ, Ferrie JJ, Xu K, Lee I, Graham TJA, Tu Z, et al. Alpha synuclein fibrils contain multiple binding sites for small molecules. ACS Chem Neurosci. 2018;9(11):2521–7. 10.1021/acschemneuro.8b00177 PMC6736640 29750499

[65] Sherer TB, Betarbet R, Testa CM, Seo BB, Richardson JR, Kim JH. Mechanism of toxicity in rotenone models of Parkinson’s disease. J Neurosci. 2003;23(34):10756–64. 10.1523/JNEUROSCI.23-34-10756.2003 PMC6740985 14645467

[66] Trist BG, Hare DJ, Double KL. Oxidative stress in the aging substantia nigra and the etiology of Parkinson’s disease. Aging Cell. 2019;8:1–23. 10.1111/acel.13031. PMC6826160 31432604

[67] Panov A, Dikalov S, Shalbuyeva N, Taylor G, Sherer T, Greenamyre JT. Rotenone model of Parkinson disease: multiple brain mitochondria dysfunctions after short term systemic rotenone intoxication. J Biol Chem. 2005;280(51):42026–35. 10.1074/jbc.M508628200 16243845

[68] Thippeswamy BS, Nagakannan P, Shivasharan BD, Mahendran S, Veerapur VP, Badami S. Protective effect of embelin from Embelia ribes Burm. against transient global ischemia-induced brain damage in rats. Neurotox Res. 2011;20(4):379–86. 10.1007/s12640-011-9258-7 21751076

[69] Naik SR, Niture NT, Ansari AA, Shah PD. Anti-diabetic activity of embelin: involvement of cellular inflammatory mediators, oxidative stress and other biomarkers. Phytomedicine. 2013;20(10):797–804. 10.1016/j.phymed.2013.03.003 23597490

[70] Kim SN, Doo AR, Park JY, Choo HJ, Shim I, Park JJ, et al. Combined treatment with acupuncture reduces effective dose and alleviates adverse effect of LD by normalizing Parkinson’s disease-induced neurochemical imbalance. Brain Res. 2014;1544:33–44. 10.1016/j.brainres.2013.11.028 PMC4180515 24321617

[71] Ordoñez-Librado JL, Anaya-Martínez V, Gutierrez-Valdez AL, Colín-Barenque L, Montiel-Flores E, Avila-Costa MR. Manganese inhalation as a Parkinson disease model. Parkinsons Dis. 2010;2011:1–14. https://www.hindawi.com/journals/pd/2011/612989/10.4061/2011/612989 PMC3010681 21209715

[72] Sakka N, Sawada H, Izumi Y, Kume T, Katsuki H, Kaneko S, et al. Dopamine is involved in selectivity of dopaminergic neuronal death by rotenone. Neuroreport. 2003;14(18):2425–8. 10.1097/00001756-200312190-00027 14663204

[73] Norazit A, Meedeniya AC, Nguyen MN, Mackay-Sim A. Progressive loss of dopaminergic neurons induced by unilateral rotenone infusion into the medial forebrain bundle. Brain Res. 2010;1360:119–29. 10.1016/j.brainres.2010.08.070 20807515

[74] Xiong ZK, Lang J, Xu G, Li HY, Zhang Y, Wang L, et al. Excessive levels of nitric oxide in rat model of Parkinson’s disease induced by rotenone. Exp Ther Med. 2015;9(2):553–8. 10.3892/etm.2014.2099 PMC4280943 25574233

[75] Bashkatova V, Alam M, Vanin A, Schmidt WJ. Chronic administration of rotenone increases levels of nitric oxide and lipid peroxidation products in rat brain. Exp Neurol. 2004;186(2):235–41. 10.1016/j.expneurol.2003.12.005 15026259

[76] Li N, Ragheb K, Lawler G, Sturgis J, Rajwa B, Melendez JA, et al. Mitochondrial complex I inhibitor rotenone induces apoptosis through enhancing mitochondrial reactive oxygen species production. J Biol Chem. 2003;278(10):8516–25. 10.1074/jbc.M210432200 12496265

[77] Joshi R, Kamat JP, Mukherjee T. Free radical scavenging reactions and antioxidant activity of embelin: biochemical and pulse radiolytic studies. Chem Biol Interact. 2007;167(2):125–34. 10.1016/j.cbi.2007.02.004 17379198

[78] Kundap UP, Bhuvanendran S, Kumari Y, Othman I, Shaikh MF. Plant derived phytocompound, embelin in CNS disorders: a systematic review. Front Pharmacol. 2017;8:1–13. 10.3389/fphar.2017.00076. PMC5326771 28289385

[79] Rao SP, Sharma N, Kalivendi SV. Embelin averts MPTP-induced dysfunction in mitochondrial bioenergetics and biogenesis via activation of SIRT1. Biochim Biophys Acta Bioenerg. 2020;1861:1–13. https://europepmc.org/article/med/31987812. 10.1016/j.bbabio.2020.148157 31987812

[80] Madathil KS, Karuppagounder SS, Haobam R, Varghese M, Rajamma U, Mohanakumar KP. Nitric oxide synthase inhibitors protect against rotenone-induced, oxidative stress mediated parkinsonism in rats. Neurochem Int. 2013;62(5):674–83. 10.1016/j.neuint.2013.01.007 23353925

[81] He Y, Imam SZ, Dong Z, Jankovic J, Ali SF, Appel SH, et al. Role of nitric oxide in rotenone-induced nigro-striatal injury. J Neurochem. 2003;86(6):1338–45. 10.1046/j.1471-4159.2003.01938.x 12950443

[82] Thomas DD, Ridnour LA, Isenberg JS, Flores-Santana W, Switzer CH, Donzelli S, et al. The chemical biology of nitric oxide: implications in cellular signaling. Free Radic Biol Med. 2008;45(1):18–31. 10.1016/j.freeradbiomed.2008.03.020 PMC2572721 18439435

[83] Weidinger A, Kozlov AV. Biological activities of reactive oxygen and nitrogen species: oxidative stress versus signal transduction. Biomolecules. 2015;5(2):472–84. 10.3390/biom5020472 PMC4496681 25884116

[84] Garry PS, Ezra M, Rowland MJ, Westbrook J, Pattinson KT. The role of the nitric oxide pathway in brain injury and its treatment – from bench to bedside. Exp Neurol. 2015;263:235–43. 10.1016/j.expneurol.2014.10.017 25447937

[85] Jankovic J, Chen S, Le WD. The role of Nurr1 in the development of dopaminergic neurons and Parkinson’s disease. Prog Neurobiol. 2005;77(1–2):128–38. 10.1016/j.pneurobio.2005.09.001 16243425

[86] Decressac M, Volakakis N, Björklund A, Perlmann T. NURR1 in Parkinson disease – from pathogenesis to therapeutic potential. Nat Rev Neurol. 2013;9(11):629–36. 10.1038/nrneurol.2013.209 24126627

[87] Jia C, Qi H, Cheng C, Wu X, Yang Z, Cai H, et al. α-Synuclein negatively regulates Nurr1 expression through NF-κB-related mechanism. Front Mol Neurosci. 2020;13:1–9. 10.3389/fnmol.2020.00064. PMC7235291 32477062

[88] Saijo K, Winner B, Carson CT, Collier JG, Boyer L, Rosenfeld MG, et al. A Nurr1/CoREST pathway in microglia and astrocytes protects dopaminergic neurons from inflammation-induced death. Cell. 2009;137(1):47–59. 10.1016/j.cell.2009.01.038 PMC2754279 19345186

[89] Oh SM, Chang MY, Song JJ, Rhee YH, Joe EH, Lee HS, et al. Combined Nurr1 and Foxa2 roles in the therapy of Parkinson’s disease. EMBO Mol Med. 2015;7(5):510–25. 10.15252/emmm.201404610 PMC4492814 25759364

[90] Volakakis N, Kadkhodaei B, Joodmardi E, Wallis K, Panman L, Silvaggi J, et al. NR4A orphan nuclear receptors as mediators of CREB-dependent neuroprotection. Proc Natl Acad Sci USA. 2010;107(27):12317–22. 10.1073/pnas.1007088107 PMC2901488 20566846

[91] Beaulieu-Boire I, Lang AE. Behavioral effects of levodopa. Mov Disord. 2015;30:90–102. 10.1002/mds.26121 25491470

[92] Müller T. Do we need a new levodopa? Neural Regen Res. 2016;11:731–2. 10.4103/1673-5374.182694 PMC4904458 27335551

[93] Müller T, Trommer I, Muhlack S, Mueller BK. Levodopa increases oxidative stress and repulsive guidance molecule A levels: a pilot study in patients with Parkinson’s disease. J Neural Transm (Vienna). 2016;123:401–6. 10.1007/s00702-016-1519-4 26880022

[94] Peelaerts W, Bousset L, Van der Perren A, Moskalyuk A, Pulizzi R, Giugliano M, et al. α-Synuclein strains cause distinct synucleinopathies after local and systemic administration. Nature. 2015;522(7556):340–4. 10.1038/nature14547 26061766

[95] Pieri L, Madiona K, Bousset L, Melki R. Fibrillar α-synuclein and huntingtin exon 1 assemblies are toxic to the cells. Biophys J. 2012;102(12):2894–905. 10.1016/j.bpj.2012.04.050 PMC3379023 22735540

[96] Drolet RE, Cannon JR, Montero L, Greenamyre JT. Chronic rotenone exposure reproduces Parkinson’s disease gastrointestinal neuropathology. Neurobiol Dis. 2009;36(1):96–102. 10.1016/j.nbd.2009.06.017 19595768

[97] Pan-Montojo F, Anichtchik O, Dening Y, Knels L, Pursche S, Jung R, et al. Progression of Parkinson’s disease pathology is reproduced by intragastric administration of rotenone in mice. PLoS One. 2010;5:1–10. 10.1371/journal.pone.0008762. PMC2808242 20098733

[98] Miyazaki I, Asanuma M. The rotenone models reproducing central and peripheral features of Parkinson’s disease. NeuroSci. 2020;1:1–14.

[99] Sampson TR, Debelius JW, Thron T, Janssen S, Shastri GG, Ilhan ZE, et al. Gut microbiota regulate motor deficits and neuroinflammation in a model of Parkinson’s disease. Cell. 2016;167(6):1469–80 e12. 10.1016/j.cell.2016.11.018 PMC5718049 27912057

[100] Morais LH, Hara DB, Bicca MA, Poli A, Takahashi RN. Early signs of colonic inflammation, intestinal dysfunction, and olfactory impairments in the rotenone-induced mouse model of Parkinson’s disease. Behav Pharmacol. 2018;29(2 and 3-Spec Issue):199–210. 10.1097/FBP.0000000000000389 29543651

[101] Klingelhoefer L, Reichmann H. Pathogenesis of Parkinson disease – the gut-brain axis and environmental factors. Nat Rev Neurol. 2015;11(11):625–36. 10.1038/nrneurol.2015.197 26503923

[102] Dodiya HB, Forsyth CB, Voigt RM, Engen PA, Patel J, Shaikh M, et al. Chronic stress-induced gut dysfunction exacerbates Parkinson’s disease phenotype and pathology in a rotenone-induced mouse model of Parkinson’s disease. Neurobiol Dis. 2020;135:1–20. 10.1016/j.nbd.2018.12.012. 30579705

[103] Piche T, Saint-Paul MC, Dainese R, Marine-Barjoan E, Iannelli A, Montoya ML, et al. Mast cells and cellularity of the colonic mucosa correlated with fatigue and depression in irritable bowel syndrome. Gut. 2008;57(4):468–73. 10.1136/gut.2007.127068 18194987

[104] Mulak A, Bonaz B. Brain-gut-microbiota axis in Parkinson’s disease. World J Gastroenterol. 2015;21(37):10609–20. 10.3748/wjg.v21.i37.10609 PMC4588083 26457021

[105] Keshavarzian A, Green SJ, Engen PA, Voigt RM, Naqib A, Forsyth CB, et al. Colonic bacterial composition in Parkinson’s disease. Mov Disord. 2015;30(10):1351–60. 10.1002/mds.26307 26179554

[106] Clairembault T, Leclair-Visonneau L, Coron E, Bourreille A, Le Dily S, Vavasseur F, et al. Structural alterations of the intestinal epithelial barrier in Parkinson’s disease. Acta Neuropathol Commun. 2015;3:1–9. 10.1186/s40478-015-0196-0. PMC4353469 25775153

[107] Metta V, Leta V, Mrudula KR, Prashanth LK, Goyal V, Borgohain R, et al. Gastrointestinal dysfunction in Parkinson’s disease: molecular pathology and implications of gut microbiome, probiotics, and fecal microbiota transplantation. J Neurol. 2022;269(3):1154–63. 10.1007/s00415-021-10567-w 33881598

[108] Huang Y, Liao J, Liu X, Zhong Y, Cai X, Long L. Review: the role of intestinal dysbiosis in Parkinson’s disease. Front Cell Infect Microbiol. 2021;11:1–13. 10.3389/fcimb.2021.615075. PMC8100321 33968794

[109] Chitra M, Devi CS, Sukumar E. Antibacterial activity of embelin. Fitoterapia. 2003;74(4):401–3. 10.1016/s0367-326x(03)00066-2 12781816

[110] Sheng Z, Ge S, Gao M, Jian R, Chen X, Xu X, et al. Synthesis and biological activity of embelin and its derivatives: an overview. Mini Rev Med Chem. 2020;20(5):396–407. 10.2174/1389557519666191015202723 31644404

[111] Feresin GE, Tapia A, Sortino M, Zacchino S, de Arias AR, Inchausti A, et al. Bioactive alkyl phenols and embelin from Oxalis erythrorhiza. J Ethnopharmacol. 2003;88(2–3):241–7. 10.1016/s0378-8741(03)00258-7 12963150

[112] Wu T, Dai Y, Wang W, Teng G, Jiao H, Shuai X, et al. Macrophage targeting contributes to the inhibitory effects of embelin on colitis-associated cancer. Oncotarget. 2016;7(15):19548–58. 10.18632/oncotarget.6969 PMC4991400 26799669

[113] Dai Y, Jiao H, Teng G, Wang W, Zhang R, Wang Y, et al. Embelin reduces colitis-associated tumorigenesis through limiting IL-6/STAT3 signaling. Mol Cancer Ther. 2014;13(5):1206–16. 10.1158/1535-7163.MCT-13-0378 24651526

[114] Colamartino M, Santoro M, Duranti G, Sabatini S, Ceci R, Testa A, et al. Evaluation of levodopa and carbidopa antioxidant activity in normal human lymphocytes in vitro: implication for oMxidative stress in Parkinson’s disease. Neurotox Res. 2015;27(2):106–7. 10.1007/s12640-014-9495-7 25355370

[115] Jodko-Piórecka K, Litwinienko G. Antioxidant activity of dopamine and L-DOPA in lipid micelles and their cooperation with an analogue of α-tocopherol. Free Radic Biol Med. 2015;83:1–11. 10.1016/j.freeradbiomed.2015.02.006 25701434

[116] Cross JM, Mercer DW, Gunter J, Miller TA. Effects of dopamine and alpha-2 adrenoreceptor blockade on L-dopa and cholecystokinin-induced gastroprotection. J Gastrointest Surg. 1997;1(3):257–65. 10.1016/s1091-255x(97)80118-7 9834356

[117] Betarbet R, Sherer TB, MacKenzie G, Garcia-Osuna M, Panov AV, Greenamyre JT. Chronic systemic pesticide exposure reproduces features of Parkinson’s disease. Nat Neurosci. 2000;3:1301–6. 10.1038/81834 11100151

[118] Cannon JR, Tapias VM, Na HM, Honick AS, Drolet RE, Greenamyre JT. A highly reproducible rotenone model of Parkinson’s disease. Neurobiol Dis. 2009;34:279–90. 10.1016/j.nbd.2009.01.016 PMC2757935 19385059

[119] Johnson ME, Bobrovskaya L. An update on the rotenone models of Parkinson’s disease: their ability to reproduce the features of clinical disease and model gene-environment interactions. Neurotoxicology. 2015;46:101–16. 10.1016/j.neuro.2014.12.002 25514659

[120] Klein C, Westenberger A. Genetics of Parkinson’s disease. Cold Spring Harb Perspect Med. 2012;2:1–15. http://perspectivesinmedicine.cshlp.org/content/2/1/a008888 10.1101/cshperspect.a008888 PMC3253033 22315721

[121] Hu D, Cui Y, Zhang J. Nervonic acid amends motor disorder in a mouse model of Parkinson’s disease. Transl Neurosci. 2021;12(1):237–46. 10.1515/tnsci-2020-0171 PMC8149914 34055392

[122] Huang N, Huang J, Zhang Y, Chen M, Shi J, Jin F. Resveratrol against 6-OHDA-induced damage of PC12 cells via PI3K/Akt. Transl Neurosci. 2021;12(1):138–44. 10.1515/tnsci-2020-0165 PMC8060978 33976931

